# Re-energising the way we manage change in healthcare: the case for soft systems methodology and its application to evidence-based practice

**DOI:** 10.1186/s12913-019-4508-0

**Published:** 2019-09-14

**Authors:** Hanna Augustsson, Kate Churruca, Jeffrey Braithwaite

**Affiliations:** Centre for Healthcare Resilience and Implementation Science, Australian Institute of Health Innovation, Macquarie University, Level 6, 75 Talavera Road, North Ryde, NSW 2109 Australia

**Keywords:** Soft systems methodology, Change management, Healthcare, Complex systems, Implementation, Intervention

## Abstract

**Background:**

Updating, improving and spreading the evidence base for healthcare practices has proven to be a challenge of considerable magnitude – a wicked, multi-dimensional problem. There are many interlinked factors which determine how, why and whether any particular implementation effort or intervention succeeds. Soft Systems Methodology (SSM), strongly grounded in systems ideas and complexity science, offers a structured, yet flexible process for dealing with situations that are perceived as problematical and in need of improvement. The aim of this paper is to propose the use of SSM for managing change in healthcare by way of addressing some of the complexities. The aim is further to illustrate examples of how SSM has been used in healthcare and discuss the features of the methodology that we believe can be harnessed to improve healthcare.

**Discussion:**

SSM is particularly suited for tackling real world problems that are difficult to define and where stakeholders may have divergent views on the situation and the objectives of change. SSM engages stakeholders in a learning cycle including: finding out about the problematical situation, i.e. the context in which the problem exists, by developing a rich picture of the situation; defining it by developing conceptual models and comparing these with the real world; taking action to improve it by deciding on desirable and feasible improvements; and implementing these in an iterative manner. Although SSM has been widely used in other sectors, it has not been extensively used in healthcare. We make the case for applying SSM to implementation and improvement endeavours in healthcare using the example of getting clinicians at the hospital level to use evidence-based guidelines.

**Conclusion:**

Applying SSM means taking account of the multi-dimensional nature of care settings, and dealing with entrenched and unique contexts, cultures and socio-political ecosystems – precisely those that manifest in healthcare. There are gains to be made in appreciating complexity and facilitating contextualization of interventions, and by approaching improvements in an iterative learning cycle.

## Background

Healthcare organisations are required to continuously update their practices to ensure that the best available care is provided to patients. However, the gap between research evidence on effective practices and practice itself is well known [1–4]. This signals a core problem: that it is notoriously difficult to update, improve and spread the evidence base for healthcare practices [5].

The success of implementation and improvement efforts depends on a myriad of factors related to the intervention itself, the process by which the intervention is being implemented, and the context in which the intervention is situated [2, 6]. However, the complexity of implementing interventions does not stop with the involvement of multifarious factors at different levels. The contextual factors that matter are likely to differ between settings as well as between interventions [7]. Making things even more difficult, these factors are interlinked and affect each other, often in unpredictable ways [8]. From this follows that every intervention, even a seemingly straight forward one, influences the overall system in which it is implemented, and the overall system influences every intervention [9]. Despite this, influencing factors are often assessed individually, assuming a linear relationship between them and the outcomes, and ignoring possible interactions between factors [8].

The intense interconnectedness of factors influencing improvement interventions calls for simultaneous consideration of all parts of the system when attempting to implement improvements—in contrast to studying or intervening in isolated parts of the system [10]. Thus, problems must be considered as they exist in the ‘real world’ [11]. In short, context matters [2, 6, 12–14], and rich, multi-faceted, structured approaches are therefore necessary for successful improvement efforts. The goal of this paper is to propose the use of SSM for managing change in healthcare by way of addressing some of these complexities. We use the example of getting clinicians at hospital level to use evidence-based guidelines, a common issue in healthcare that has proven to be challenging and largely influenced by contextual factors [15, 16]. An additional aim is to present some illustrative examples of how SSM has been used in healthcare and to discuss the features of the methodology that we believe can be harnessed to improve healthcare.

## Main text

### Soft systems methodology - a systems approach to improvement

A systems perspective assumes that systems are wholes, not readily decomposable, comprised of interdependent components with flexible, porous boundaries. The interacting components (artefacts, buildings, equipment, individuals and groups) combine in unanticipated ways over time, behaving and interacting dynamically [11]. Designed to encapsulate such complex stochastics, Checkland and colleagues [17, 18] developed Soft Systems Methodology (SSM). SSM is based on systems ideas and is described as a structured, yet flexible, process for dealing with situations that are perceived as problematical and in need of actions to improve the situation [19]. SSM has a broad application but may be particularly suited to messy problems where the problem situation is hard to define, where stakeholders have divergent views about the situation and the objective of change [19] and when attempts to improve things have failed [20]. SSM is a comprehensive methodology and has a number of concepts and tools developed explicitly for it. Table 1 provides an overview and descriptions of these.

**Table 1:** Glossary of terms and acronyms used in SSM

SSM – Abbreviation for Soft Systems Methodology	
Rich picture – Exploration of the problematical situation and description of it by making drawings of the situation, including the various stakeholders’ roles, and the structures and processes as well as the relationships between these.	
Worldview – Underlying assumptions about the world, also known as *weltanschauung* in SSM.	
Human activity system –The meaning of a system in SSM is a set of human activities aiming to achieve a purpose.	
Purposeful activity – Defined by a transformation process, i.e. an input being transformed to an output, within the scope of a worldview.	
Purposeful activity model (PAM) – A conceptual model for one or more aspects of the problematical situation outlining a set of purposeful activities relevant to the situation. The model is a set of linked activities that together makes up a purposeful whole.	
Root definition – A statement describing the human activity system to be modelled.	
CATWOE –A reminder to consider the following information about the human activity system:	
Customers –The beneficiaries or victims affected by the problematical situation and the improvement intervention.	
Actors –The individuals involved in performing the improvement intervention.	
Transformation – The change process.	
Worldview – Underlying assumptions that makes the improvement intervention worthwhile and important.	
Owners – The actors that are responsible for the improvement intervention and who decide whether it will be implemented or not.	
Environmental constraints and enablers – The contextual factors that may influence the problematical situation and the improvement intervention.	
The PQR-formula – A formula useful for defining the root definition. It is applied by answering the questions: what should be done (P), how it should be done (Q) and why it should be done (R).	
Three E’s – Criteria for assessing the outcomes of the improvement intervention, including:	
Efficacy – does the intervention produce the intended outcomes?	
Efficiency – is the improvement being achieved with minimum use of resources?	
Effectiveness – does the intervention help achieve some higher-level or longer-term aim?	

Explanations are based on Checkland and Poulter [19] but interpreted by us and adapted to a language more often used in relation to implementation and improvement science

A fundamental idea behind SSM is that the process of inquiry into the complexity of the ‘real world’ can be simulated as a learning process. The learning process goes from finding out about the problematical situation to defining it, and taking action to improve it. ‘Real world’ in SSM language refers to the unfolding and interacting flux of events and ideas experienced as everyday life [21] and this is distinguished from the system thinking world in which conceptual models to learn about the ‘real world’ and how to improve the situation are created. An important aspect of SSM is that it recognizes peoples’ diverging underlying assumptions about the world, i.e. their disparate worldviews. These different worldviews affect their understanding of the problematical situation and potential solutions. Thus, any one-size-fits-all solution, or even a uni-dimensional view of what the problem is, will never approximate the complexity of the real world. For SSM, individuals will always try to act purposefully but will proceed from their own perspective and thus will behave differently from other actors [18, 19].

SSM invites relevant stakeholders in a given context to participate in the process of improvement, taking account of their differing perspectives. This, in turn, has the effect of engaging them in change processes and moving towards a model predicated on continual improvement rather than treating stakeholders as the implementation arm of a change project, the subjects in an intervention, or barriers to, or resistors of, change. The SSM learning and change management approach is well defined by Checkland and colleagues (e.g. [18, 19]), but we summarise it here into the four activities of the SSM process:

#### Finding out about the problem, including culturally and politically

As a first step, the focus should be on understanding the problematical situation, i.e. the circumstances in which the problem may exist, rather than the problem itself. The SSM process starts with at least one stakeholder perceiving that things could be better than they are or that there is some perceived problem requiring attention. This does not necessarily mean that all relevant stakeholders perceive it to be a problem or perceive the situation in the same way. Thus, in SSM it is important to gain perspectives from different stakeholder groups, e.g. different clinical staff groups, managers and patient representatives.

Different methods and information sources may be used to gain an understanding about the situation. More interactive methods such as focus groups or workshops can facilitate the creation of a common understanding about the situation and the objective of change. However, focus groups, especially if performed with mixed stakeholder groups, pose a challenge when it comes to power structures. Groups of members with differing levels of power (e.g. care providers and patients) imply the risk of individuals with lower levels of power participating to a lesser extent [22]. A basis of this activity, and in SSM in general, is that no perspective is more or less important and that minority opinions or opinions not in agreement with the official line should not be disregarded. Also, participation from all relevant stakeholders should be facilitated and encouraged. Thus, attention needs to be paid to the power structures in the SSM process just as in focus groups. This requires a skilled facilitator and sometimes other methods to collect stakeholders’ views about the situation, e.g. interviews, may be better even if this decreases the possibilities for debate.

In addition to eliciting the perspectives from different stakeholders, it is also important to investigate different perspectives of the problematical situation. This involves analyses of: 1. the intervention, including the actors involved, 2. the socio-cultural context including roles, norms and values and 3. existing power structures.

**Fig. 1:**
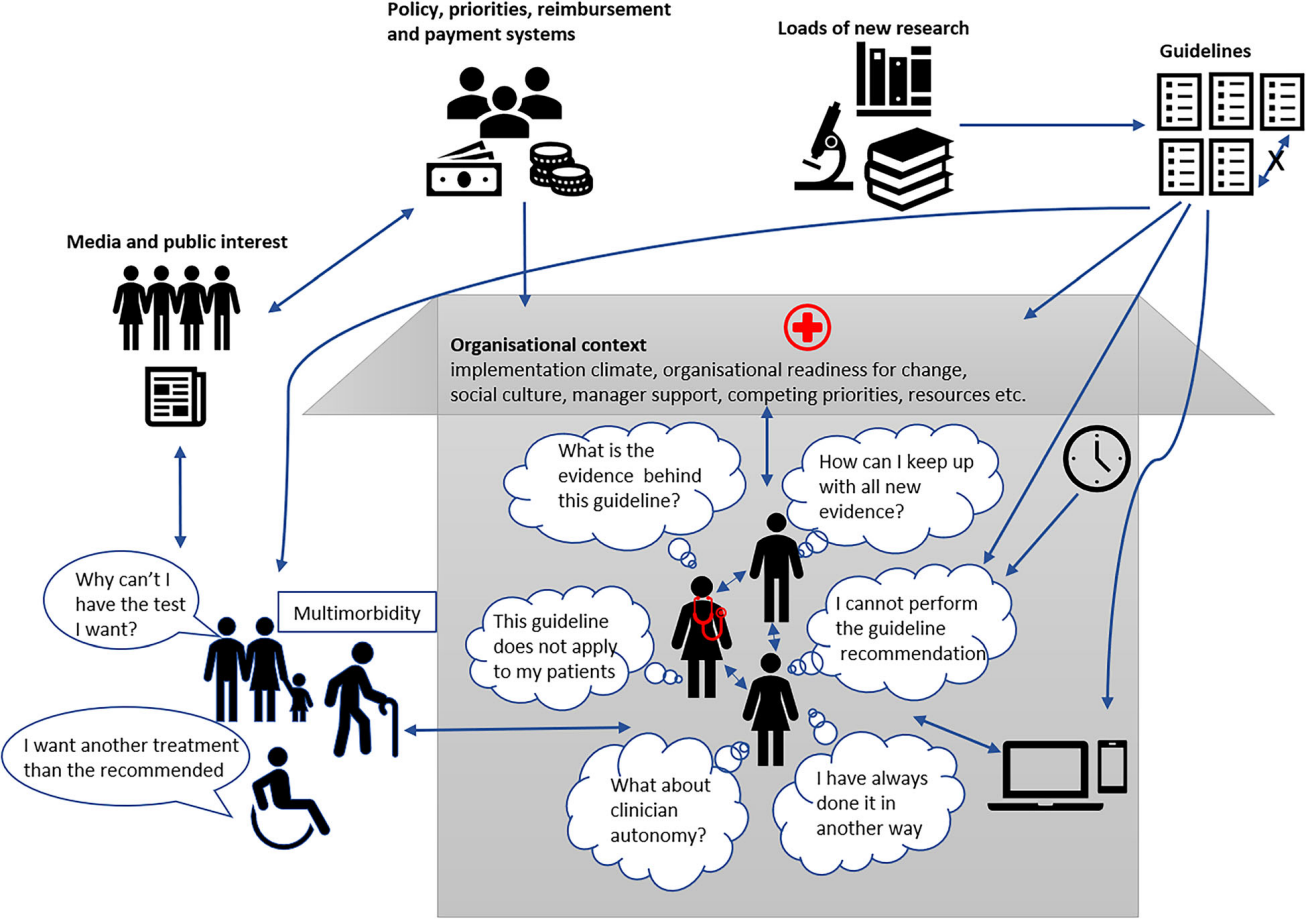
Example of a rich picture. *Legend:* The picture illustrates the interlinked relationships influencing implementation of evidence-based guidelines in a hospital. The picture is based on Figure 3.2 in Greenhalgh [10] and the authors’ own experience in implementation science. N.B. all conceptualisations are a simplification of the real world and we do not claim that all potentially important factors are illustrated in the picture

This activity helps to define the problematical situation, allowing different perspectives to be considered. The gathered information, e.g. from interviews, focus groups and documents, is used to develop a rich picture, which describes the problematical situation in drawings or diagrams and helps to elucidate the links between different factors, processes and structures. We have come to think of this as the *“a picture tells a thousand words”* activity (Fig. 1: Example of a rich picture).

#### Formulating relevant purposeful activity models (PAMs)

This activity involves creating a conceptual model of one or more aspects of the problematical situation outlining a set of purposeful activities relevant to the situation. A model can only be based on a single declared worldview and thereby represents one way of looking at a complex reality. The model is not intended to be a perfect model to be implemented but used as a basis for discussion and learning about the problem situation and potential ways to improve it. This, we label the *“simulation-modelling of the world”* activity.

SSM theory articulates several tools for use by its adherents (see Table 1 for terms) in order to facilitate the formulation of PAMs. One such tool is the *root definition,* which is a statement describing the activity system to be modelled. Formulation of root definitions can be helped by using the *PQR formula* which answers the questions: what should be done (P), how should it be done (Q) and why should it be done (R). In SSM language, the task here is to do P by Q in order to achieve R.

A PAM, and the learning and discussions based on the model, should include a specific set of information in order to be comprehensive enough to guide further work. This is facilitated by another tool in SSM, summarised by the *CATWOE* mnemonic. The C stands for *customer* (e.g. in our case, this might typically be patients) and represents the group of beneficiaries, or victims, who are affected by the system’s activities. The A is for the *actors* (e.g. healthcare professionals) that are responsible for carrying out the main activities of the system, i.e. to make the envisaged change. The T depicts for *transformation* and represents the process by which inputs are converted to outputs. The W stands for *worldview* and represents key stakeholders’ underlying assumptions about why the transformation is important. The O is the *owner* of the system and includes people and roles that can change or stop the transformation process (e.g. healthcare professionals, administrators, policymakers) and the E represents the *environmental constraints and enablers*, i.e. contextual factors, that influence the PAM. It is worth noting that people and roles can fall into more than one group.

**Fig. 2:**
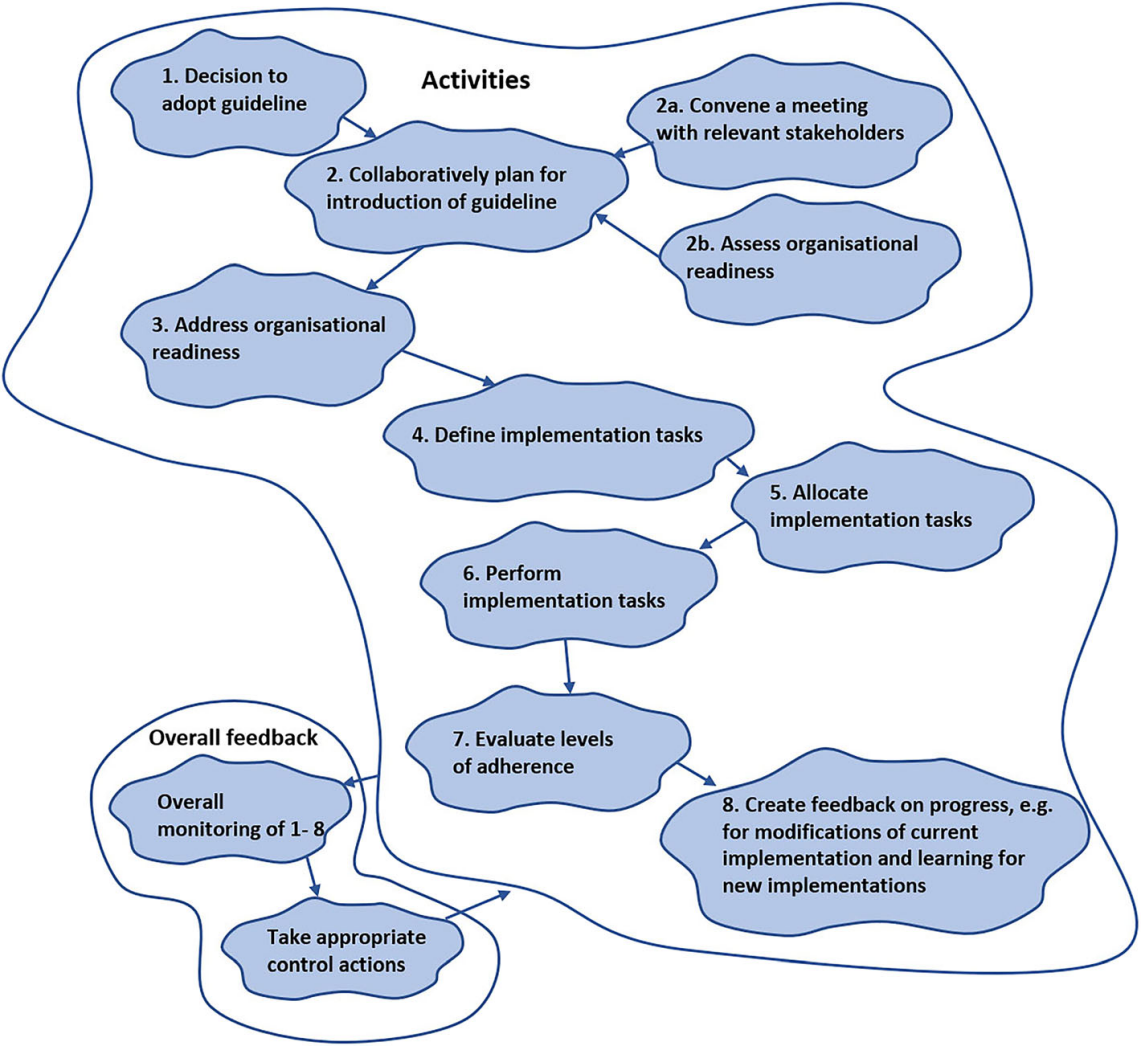
An illustrative example of a PAM. *Legend:* The PAM outlines a generic process for implementation of evidence-based guidelines into practice in a hospital setting

It is useful to think about how to assess the outcomes of the PAMs and formulate criteria for *efficacy, efficiency and effectiveness* (the three Es in SSM language). This helps to guide continuous monitoring of the progress of an intervention which in turn provides information enabling relevant control actions to be taken to improve the system activities and the outcomes. Altogether, the information gained from developing the root definition, PQR, CATWOE and the three E’s (Table 2: An illustrative example of the application of SSM tools) is used to create a relevant PAM (Fig. 2: An illustrative example of a purposeful activity model).

**Table 2:** An illustrative example of the application of SSM tools

Root definition	
A system to implement evidence-based guidelines, by using a hospital-level generic process, to provide patients with best available and equitable care, owned and managed by hospital administrators, healthcare professionals and policy makers.	
PQR formula – Do P by Q in order to achieve R	
P Implement evidence-based guidelines	
Q by using a hospital-level generic process	
R in order to provide patients with best available and equitable care.	
CATWOE	
Customers	Patients, healthcare professionals
Actors	Healthcare professionals, administrators
Transformation	Guidelines implemented and adhered to
Worldview	Evidence-based guidelines support best available and equitable care being delivered to patients
Owners	Policy makers, administrators, healthcare professionals
Environmental	Inner and outer context - multiple and interacting factors constraints and enablers
Three E’s – Criteria for assessing the outcomes of the improvement intervention, including:	
Efficacy – does the intervention lead to higher adherence to guidelines?	
Efficiency – is the improvement being achieved with minimal use of resources?	
Effectiveness – does the intervention help achieve higher quality of care for patients?	

An illustrative example of a Root definition, a PQR formula, CATWOE and the three E’s applied to the problem of getting evidence into practice using evidence-based guidelines

#### Debating the situation, using the models

In this third activity, the information gained from developing the rich picture together with the PAM is used to organise a discussion about potential improvements. The simulated model of the world helps illuminate differences between the way the stakeholders are constructing the world (the PAM), and the problem situation, which enables the questions that will ultimately lead to change. The simulated model should not be viewed as a perfect model but simply as a device to structure discussion about improvements. The focus should be on both:

- changes which would improve the current situation that are both desirable and culturally feasible
- accommodations between conflicting interests amongst stakeholders which will enable improvements to be made

The aim of this third activity is to find changes that can lead to improvements and that are contextually and culturally feasible in the specific situation. It also aims to acknowledge the conflicting views in health care – doctors, nurses, allied health practitioners, managers, patients and policymakers all differ in their perspectives from each other, and to accommodate these divergent views. This third activity, drawing on the idea from resilient health care [23], we name the *“bridging the world-as-imagined so it is in line with the world-as-done”* activity.

#### Taking action to bring about improvement

This activity involves identifying opportunities for improvement based on the previous activities. It then proceeds to testing changes as a basis for further learning amongst stakeholders involved in the change.

The testing is done iteratively to challenge and adapt the improvement intervention. This iterative testing is facilitated through monitoring of progress and by taking control actions based on this. We call this the *“change-in-context, realised”* stage.

**Fig. 3:**
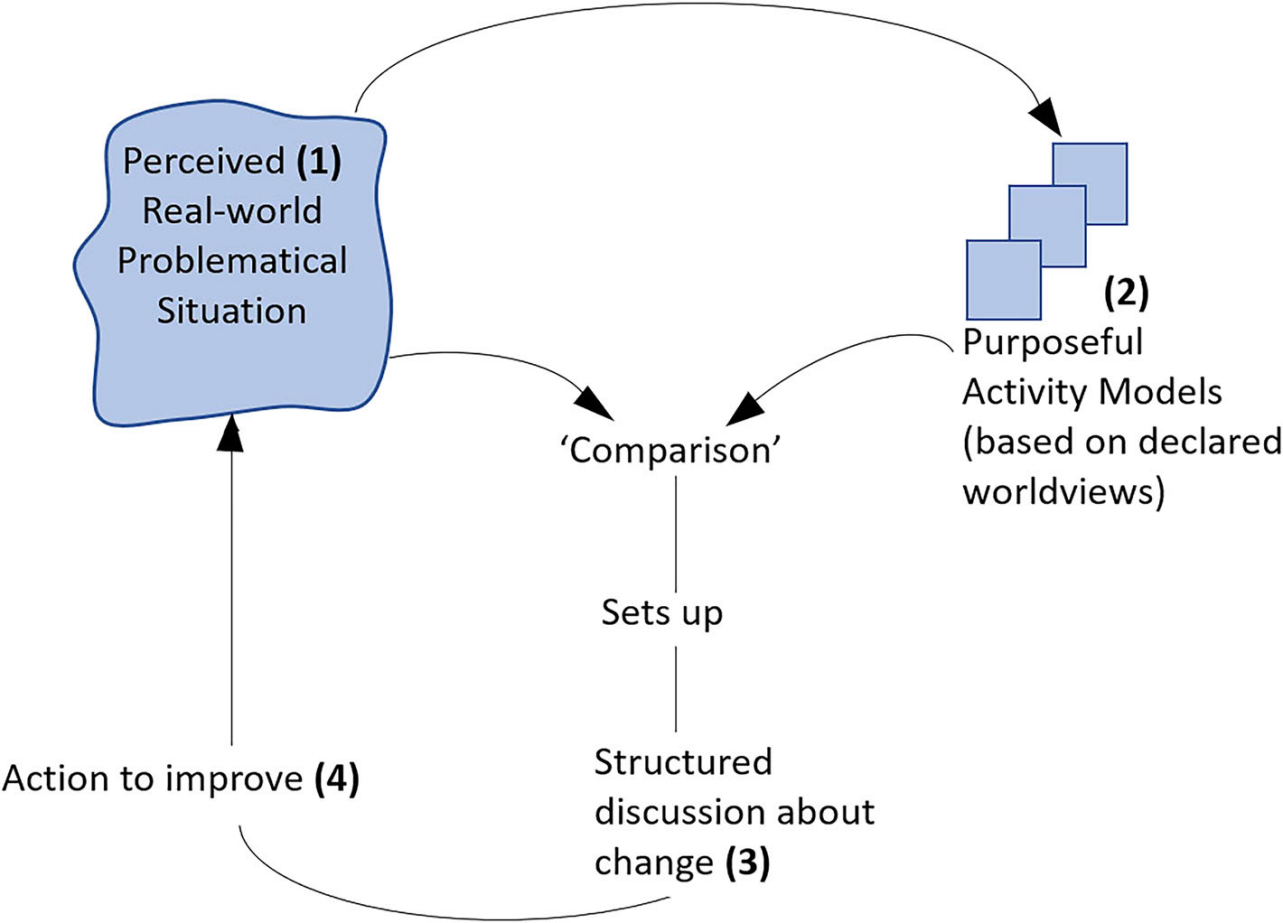
A generic SSM learning cycle. *Legend:* Source: Checkland and Poulter [19]. Permission granted by John Wiley and Sons for use of this image. Licence number: 4390591134436

In SSM thinking, the process does not stop when the fourth activity is “completed”—because there is no such thing as being finished in a complex system such as that which delivers care to patients. SSM is a continuous learning process and since services and organisations are under continuous development and variables are in constant motion, problem-solving processes and improvement efforts must be flexible and accommodating to real world fluidity and dynamism Fig. 3. Illustrates a generic SSM learning cycle with all four activities outlined.

### Application of SSM in healthcare

SSM has been used in a range of different fields [24]. However, it has lagged in healthcare, for reasons that are not completely clear. We found 871 articles on SSM in the multidisciplinary database, Scopus, but only 21 empirical studies conducted in healthcare in PubMed, the health and medical database.

The identified papers show that when SSM has been applied in healthcare, it has been used as a structured way of analysing problematical situations alone (e.g. [25]), for a combination of problem analysis and suggestion for and/or development of improvement interventions (e.g. [26–30]) and policies (e.g. [31, 32]), as well as for the evaluation of interventions (e.g. [33, 34]). When it has been used, it has been applied in several different healthcare settings including: acute care, community care, child and adolescent care, emergency care, mental health, and palliative care. Table 3 provides some specific examples of how SSM has been used for healthcare improvements. The identified studies illustrate SSM’s flexibility and versatility—it can be useful for a range of different problems in healthcare as well as in a range of different healthcare contexts. Furthermore, the examples show that SSM has been applied in different ways, e.g. using different data collection methods and SSM tools and involving stakeholder groups to a varying extent.

**Table 3:** Illustrative examples of how SSM has been applied in healthcare

Type of problem situation	Setting	Data collection and no. of stakeholder groups consulted	Featured SSM tools	Solution	Outcome
Reducing tensions in the patient discharge process [30, 35]	Hospital, UK	Interviews, workshop 17 stakeholder groups	Rich picture, CATWOE, Root definition, PAM, comparison of PAM and the ‘real-world’ situation	Three approaches for improving the process were recommended.	Implementation of the three improvements reduced total length of stay by 67% from 55.8 days to 18.6 days for the patients studied.
Policy -implementation of diabetes National Service Framework (NSF) [32]	National Health Services (NHS), UK	Interviews 2 stakeholder groups	Rich picture, CATWOE, Root definition, PAM, comparison of PAM and the ‘real-world’ situation	Issues relating to human communication, information provision and resource allocation were identified and desirable and feasible changes to achieve a more effective NSF implementation were proposed.	N/A
Development of context-appropriate informatics tools [27]	Ambulatory care, USA	Interviews, observations 7 stakeholder groups	Rich picture	A framework of ten guidelines for the design and implementation of health information technology solutions for chronic disease care were developed.	N/A
Complex care pathway [29]	Multiple sectors, UK	Interviews, systematic review, online discussion forum, national care audit datasets 12 stakeholder groups	Rich picture, CATWOE, Root definition, PAM	Evidence-informed recommendations for service improvement for congenital heart disease services were developed.	A coherent set of targeted recommendations for service improvement fed into national decisions about service provision and commissioning.
Asthma emergency care [26]	Emergency department at a hospital, UK	Interviews, focus groups 3 stakeholder groups	CATWOE, Root definition	An asthma patient passport aimed to increase patient’s confidence in their ability to communicate their needs while in severe distress was developed.	N/A
Child and adolescent mental health policy development [31]	Mental health policy, Belgium	5 stakeholder events (in-depth interviews, workshops and discussion tables), narrative review, a content analysis of policies in 3 other countries/regions At least 7 stakeholder groups	Rich picture, Root definition, PAM	Ten strategic recommendations for how to lay down the contours of a more effective system were proposed	N/A
Evaluation of a telehealth intervention for children with neurogenic bladder [33]	Hospital, telehealth intervention, UK	Interviews, observations, survey 3 stakeholder groups	CATWOE, Root definition	SSM was used as part of the evaluation of a telehealth intervention for urinalysis monitoring for children with neurogenic bladder. No solution was proposed.	N/A

However, the studies also highlight limitations in the empirical evidence for the use of SSM in healthcare. SSM has most often been used to structure a problem and to make recommendations for improvements but to a lesser extent to take action to improve and evaluate the outcomes from this. Of the identified studies, only three [29, 30, 36], mentioned implementation of the proposed improvements and of those only two presented the outcomes of the implementation in subsequent papers [35, 37]. It seems that SSM has been considered most useful in the initial stages of an improvement process, when defining the problem situation and exploring potential solutions and less useful in the process of putting these improvement suggestions in place. Without the next step of evaluating the implementation and outcomes of the improvements it is difficult to fully assess the usefulness and effectiveness of SSM for healthcare improvement.

### How can SSM be harnessed to improve healthcare?

With growing understanding of healthcare as a complex adaptive system [38, 39] not amenable to linear, top-down change strategies [11], it is timely to revisit the potential importance, and utility, of SSM. Because of failures of the past (many change strategies fail, and many more fall short of their sponsors’ intentions) most change experts will agree that we must move towards a learning system—one that applies more multi-faceted, systems-receptive change models, and evaluates progress across time (e.g. [10, 38, 40–43]). We propose that using SSM as this structured, multifaceted approach has the potential to facilitate contextually adapted improvements in healthcare by: involving stakeholders affected by change and with expertise about the local context, facilitating contextualization of improvement interventions to the local context, taking a systems approach to assess and address the nominated situation, and by approaching improvements in an iterative learning cycle. Below we outline our proposed key principles for the use of SSM in healthcare in future.

#### Participation

Any successful intervention requires individuals to change behaviour in some way [44]. As Greenhalgh et al. [2] expressed it, “People are not passive recipients of innovations. Rather (and to a greater or lesser extent in different persons), they seek innovations, experiment with them, evaluate them, find (or fail to find) meaning in them, develop feelings (positive or negative) about them, challenge them, worry about them, complain about them, “work around” them, gain experience with them, modify them to fit particular tasks, and try to improve or redesign them—often through dialogue with other users”.

This means that the individuals involved can make or break an intervention and that it is vital to include them in the process. We must not treat them as subjects but *participants.* This is in line with the emergence of partnership research [45] and models of collaboration, and co-production of knowledge in healthcare which emphasise that knowledge is generated within its context of use [46–48]. A core component of SSM is that it proposes a collaborative approach to problem solving and change management. It explicitly seeks to collect different views of a problematical situation (activity 1), as well as involving stakeholders in improving the situation (activities 2–4). This helps to highlight varying views of the situation, the dimensions of the intervention, and to take different perspectives into consideration. By highlighting individuals’ beliefs, perceptions and attitudes, levels of readiness for change can be detected and addressed to improve the likelihood of successful outcomes [49, 50].

#### Contextualization and taking a systems approach

In the case we make above, context matters, and an intervention that is adapted to fit the local circumstances is more likely to be successful and sustained [2, 40, 51, 52]. Thus, there are good reasons to consider how improvement interventions could be contextualized. SSM facilitates this in two ways. First, the participatory approach involves different stakeholders with unique context knowledge who use this knowledge to analyse the problematical situation and contribute to change management. Second, the systems thinking associated with SSM implies that the whole system is taken into consideration rather than looking at individual components in isolation. This facilitates alignment between different parts of the systems and decreases the risk of making changes that have unintended and unwanted consequences for other parts of the system. Similarly, it can help to illuminate the processes and systems that are already in place and working, in order to take advantage of these when making improvements, e.g. by linking the improvements to these processes and systems [42].

#### Continuous adaptations and learning

The dynamic and changing nature of healthcare organisations and the context in which they subsist necessitate continuous adaptation and refinement of interventions [40]. Yet another argument for continuous adaptation is that it is often impossible to take every potential problem and influencing factor into consideration prior to implementation. This calls for a move away from the traditional methods of evaluating interventions where processes and outcomes are evaluated months or years after initial implementation, towards the use of rapid feedback loops to assess intervention progress [40–42]. SSM addresses this by engaging participants in an iterative process of assessing their local context, making improvements and then doing things again. Within the SSM paradigm, the learning process is continuously monitored to assess progress and problems so that relevant control actions can be taken to refine or change the implementation and the intervention.

The process of SSM also has the potential to facilitate organisational learning. By involving different stakeholders, knowledge sharing and knowledge creation as well as the development of shared meaning and understanding across individuals and groups are enabled [53, 54]. The involvement of organisational members in analysing, developing and testing improvements can facilitate a culture that supports experimentation, where people are comfortable with questioning current practices and encouraged to explore new ideas and innovations [53]. Finally, by engaging stakeholders in the improvement process they learn about how to use a structured approach to making improvements, which can be applied in future improvement efforts.

### SSM in relation to other change management and implementation approaches

SSM entails both similar and unique features when compared with other approaches to organisational improvement. One example is the investigation of the context in which the problem situation is located, an important first activity in SSM. In this sense it is similar to implementation determinant frameworks (e.g. [2, 6]) and process models (e.g. [55]). However, these approaches generally provide guidance, e.g. in the form of lists, for what factors may be important and should be assessed, which is not specified in SSM. SSM on the other hand uses pictures or diagrams to explore the context so that links between different parts can be identified. This may help avoid seeing influencing factors and parts of the system as separate from each other.

SSM also has similarities to other approaches when it comes to managing change in an iterative learning cycle. For instance, Plan-Do-Study-Act [56], Dynamic Sustainability Framework [40] and Normalization Process Theory [57] all entail this component and few scholars or practitioners dealing with change in healthcare, believe that it is a straightforward process. What distinguishes SSM is that it uses system thinking to create models that can be used to learn about the situation in need of improvement and helps to explore and decide on feasible and desirable changes.

Another difference is that while implementation approaches are focused on describing or guiding the implementation process, understanding influences of implementation and evaluating implementation [8], SSM is more focused on the problem structuring. As such, SSM may be especially suited for ill-defined problems and can help assist in defining the intervention to be implemented and therefore contribute to the step before actual implementation. Thus, it may be used to complement implementation approaches.

### Limitations

We have argued that SSM can be used to engage stakeholders in a collaborative process of making contextualized improvements and have outlined key principles for this. As to limitations, while SSM involves aspects that are important for implementation, e.g. participation, consideration of contextual factors and continuous evaluation [6], it provides little guidance for how to perform the last step, i.e. taking action to improve except for making improvements in an iterative way. This may be one reason why the identified studies mainly applied SSM as a way to structure problems and come up with suggestions for improvements and to a much lesser extent for implementation of the improvement actions. Another limitation is the relatively low number of empirical studies which makes it challenging to draw conclusions about the impact of SSM in healthcare.

The technicalities of SSM can make it difficult to appreciate and apply, especially for people who are not used to systems modelling or SSM language. Application often requires facilitation by an SSM expert (from inside or outside of the organization) who is familiar with the process and SSM tools and mechanisms [58, 59]. Thus, SSM application will often require experience or technical support. Furthermore, since it is a participatory approach it requires the organisation and the individuals in it to be invested in the process for it to be worthwhile. To ensure support and build trust and understanding with involved practitioners it is important to secure allocated time, arenas for interactions as well as skills in project management and communication [60]. Finally, we do not provide a detailed guide for how to use SSM. For this we refer to the books by Checkland and colleagues on the topic (e.g. [19]).

## Conclusion

Complex systems like healthcare require multi-faceted solutions. The time for attempting change via unsophisticated, linear, top-down means in complex health settings is surely over. We have put forward the case for using SSM to re-energise the way we manage change in healthcare and highlighted participation, contextualization, taking a systems approach, factoring in complexity thinking, and embracing continuous adaptation and learning as key principles for change which can be facilitated by applying SSM logic, tools and approaches.

## Data Availability

Data sharing is not applicable to this article as no datasets were generated or analysed during the current study.

## Abbreviations

CATWOE: Customer, actor, transformation, worldview, owner, environmental constraints and enablers
PAM: Purposeful activity model
PQR: What, how, why
SSM: Soft Systems Methodology
Three Es: Efficacy, efficiency and effectiveness
